# Collective heart rate telemetry reveals frequent cardiac responses to social interactions but stronger responses to predation

**DOI:** 10.1098/rsbl.2026.0478

**Published:** 2026-11-11

**Authors:** James A. Klarevas-Irby, Hanja B Brandl, Damien R. Farine

**Affiliations:** ^1^ Division of Ecology and Evolution, Australian National University, Canberra, Australia; ^2^ Department of Migration, Max Planck Institute of Animal Behavior, Radolfzell, BW, Germany; ^3^ Mpala Research Centre, Nanyuki, Laikipia, Kenya; ^4^ Department of Collective Behavior, Max Planck Institute of Animal Behavior, Konstanz, BW, Germany; ^5^ Center for the Advanced Study of Collective Behaviour, University of Konstanz, Konstanz, BW, Germany; ^6^ Department of Ornithology, National Museums of Kenya, Nairobi, Kenya

**Keywords:** collective behaviour, heart rate, sociality, predation, physiological costs

## Abstract

Animals form groups to overcome environmental challenges, such as reducing the costs of predation. However, forming groups introduces new challenges and costs. For example, there is growing evidence that social interactions can be a source of physiological stress. What remains unknown is how often individuals experience these different stressors and their relative impact. Here we use a novel approach, collective heart rate (HR) telemetry, to capture the occurrence of likely socially driven and predator-related acute cardiac responses. We find that likely predator-related responses (when the majority of the group experiences a simultaneous spike in HRs) induce stronger behavioural and cardiac responses, but that socially driven responses (where two to three individuals experience a spike) occur much more often. Specifically, individuals experience 11 times more socially induced than predator-related HR spikes. These findings highlight the emergent trade-offs that arise when animals form groups.

## 1. Introduction

Animals face numerous environmental stressors, with predation being particularly pervasive. Predation is a key driver underlying the evolution of group living [1], as groups can dilute individual-level risk [2,3], better detect predators (i.e. the ‘many eyes’ hypothesis [4]) and make it more difficult for predators to target individual prey (i.e. the ‘confusion effect’ [5,6]). While predation risk may not always trigger a stress response (especially if animals mitigate threats, e.g. by avoiding riskier areas [7]), direct predator exposure can activate stress systems [8], particularly the sympathetic nervous system [9,10]. For example, elk (*Cervus elaphus canadensis*) showed elevated heart rates (HRs) in response to fresh faeces of cougars (*Puma concolor*) and grey wolves (*Canis lupus*) [11], while horses showed a higher HR during and after playbacks of grey wolves and Arabian leopards (*Panthera pardus nimr*) [12]. Studies commonly quantify how often predators encounter prey or prey adaptations to risk (such as where, when and with whom to forage [13]), but these measures do not indicate how often individual prey actually experience acute stress.

We now know that conspecifics can also act as a source of physiological stress. Nesting penguins (*Pygoscelis adeliae* and *Eudyptes robustus*) showed increased HR when conspecifics were nearby [14,15], while European starlings (*Sternus vulgaris*) showed a clear temporal spike (i.e. a rapid increase) in HR when introduced to new individuals [16]. Even within social groups, where members are otherwise tolerant to each other, stress can arise through agonistic social interactions, such as chases and physical fights [17], driving acute physiological responses [18]. For example, free-living greylag geese (*Anser anser*) experienced higher HR with more intense and longer agonistic social interactions [18], and bystanders with strong social connections to a recipient also showed elevated HR [19], indicating social stress transmission [20]. Similarly, group-living sows showed stronger HR increases following physical contact versus threats [21]. Yet, data on how often prey face different sources of stress under natural conditions, and the relative intensity of different exposure events, are scarce.

In this study, we aim to evaluate two hypothesized sources of physiological costs: social interactions and predation threats. While there is support for each of these hypotheses independently, here we advance our understanding of their relative importance by comparing both the frequency of these two stimuli and the magnitude of the physiological responses they elicit, using a common metric (acute changes in HR). Specifically, we use a fine-scale collective physiology approach measuring HR simultaneously across members of a wild vulturine guineafowl (*Acryllium vulturinum*) group. Vulturine guineafowl are large (1.3–1.8 kg), long-lived, largely terrestrial and live in stable, cohesive social groups (individuals are typically within 30 m of one another [22]). They face many predators, both aerial (e.g. martial eagles *Polemaetus bellicosus*) and terrestrial (e.g. jackals *Lupulella mesomelas*), and respond readily to alarm calls from conspecifics and heterospecifics (e.g. white-bellied go-away-birds *Crinifer leucogaster*). Alarms or attacks prompt strong, coordinated responses in guineafowl with the group fleeing to cover and vocalizing loudly [23]. We expect that such events would affect most group members simultaneously, especially given the highly cohesive nature of groups. Social interactions, by contrast, typically only affect a subset of group members [17–19]. We record simultaneous HR spikes (rapid increases in HR to values above baseline) across group members, assigning events as likely social interactions when two to three individuals show a simultaneous spike or as likely predator events when the majority of individuals show a spike. From these data, we test two predictions: (i) that responses to social interactions occur more frequently than responses to likely predation threats, and (ii) that likely predation threats elicit stronger physiological responses than those arising from social interactions.

## 2. Methods

### (a) Heart rate data collection

We implanted electrocardiogram (ECG) loggers into 13 vulturine guineafowl within a social group of 28 individuals [22] that form part of a long-term study [17,22,24–28] at the Mpala Research Centre, Kenya. These high-resolution ECG loggers (ECG-tag 1AA2, e-obs GmbH, Grünwald, Germany) have two electrodes that directly measure the electrical signals corresponding to heartbeats. Loggers were sterilized using ethylene oxide and implanted into the thoraco-abdominal cavity of six males and seven females in our study group between 25 February and 2 March 2021. Birds were anaesthetized with isoflurane 5% and oxygen (1–3 l min^–1^); butorphanol 1.5 mg ml^–1^ was injected into the pectoral muscle to provide analgesia during surgery, and Ringer's solution (20 ml kg^–1^, SC) was injected for fluid maintenance. An incision was made in the linea alba (approx. 2 cm length) where the ECG loggers were implanted. We placed the longer electrode close to the heart, and after confirming visually (QRS complex) that the live ECG signal was of good quality, the logger was fixed to the abdominal wall using an absorbable suture (Monosyn® 4/0, B. Braun AG, Melsungen, Germany). Postoperative pain was managed with meloxicam IM 0.5 mg kg^–1^ and marblofloxacin 5 mg kg^–1^ IM was injected as infection prophylaxis. Loggers weighed 25 g or about 2% of the body weight of the smallest vulturine guineafowl that we tagged (1.3 kg; mean adult weights: females 1410 g, males 1625 g).

We used data from 10 March to 12 July, 2021, giving individuals time to recover from implantation. We programmed ECG tags to operate every 4th day over the most-active portion of the day (06.00 am to 12.00 pm [22]). ECG tags had a fixed battery life, so we used a battery lifetime calculator provided by e-obs to design our data sampling strategy to maximize sampling frequency, the coverage of the periods in which birds were predominantly active and the duration of data collection. This resulted in collecting a 6 s burst of data at 180 Hz with a 14 s gap between bursts. We used data from 06.30 am (after birds left the roost) until the end of the recording period, which became earlier over the study owing to clock drift (see [22] for details on how we corrected for drift). Data were downloaded the evening after data collection using a VHF antenna. HR were extracted from the data using a custom-made peak detection algorithm [22], obtaining HRs for each 6 s data window. Finally, we used pre-06.00 am data (owing to clock drift) to extract a value for resting (baseline) HR of each individual on each day of data collection.

### (b) GPS data collection

We fitted solar-powered GPS loggers (e-obs 15 g solar) to all vulturine guineafowl that we trapped from the focal social group, including the HR birds [22]. Tags were fitted using backpack harnesses with Teflon ribbon and elevated using 24 mm of TPE matting. The total weight of the GPS logger plus backpack and harness was less than 2% of the body weight of all individuals. The combined weight of all materials (ECG logger, GPS logger, harness and rings) was less than 4% of body weight (mean 3.3%, range 2.8–3.8%).

We programmed GPS tags to collect data for all daylight hours when birds were active (06.00 am to 19.00 pm) on the same days that ECG loggers were recording. This maximized battery charge and ensured that all tags collected data simultaneously [29] at a continuous 1 Hz rate (one GPS location per second) while ECG data were being collected. Data were downloaded at night using a VHF antenna.

GPS tags have low positional error, typically less than 5 m but much lower when collecting at higher sampling frequencies. The relative error between tags collecting data simultaneously is also lower than the absolute error [30]. Previous testing of the relative error suggests that distances between individuals were accurate to within 1 m for over 95% of the data [24].

### (c) Identifying heart rate spikes

We defined spikes as individuals exhibiting a sudden, strong HR increase. Specifically, spikes corresponded to increases in HR from one burst of data to the next that exceeded the 95th percentile of all changes for that individual (figure 1A). Because these spikes could include returns from low HR values, we only kept those where the resulting corrected HR value exceeded the sum of that individual's daily median HR and the 95th percentile of change values (see supplementary material for sensitivity analyses on this value). Thus, spikes captured only cases where an individual experienced a strong increase in HR from one window to the next and where that increase resulted in them having a high HR value.

**Figure 1.**
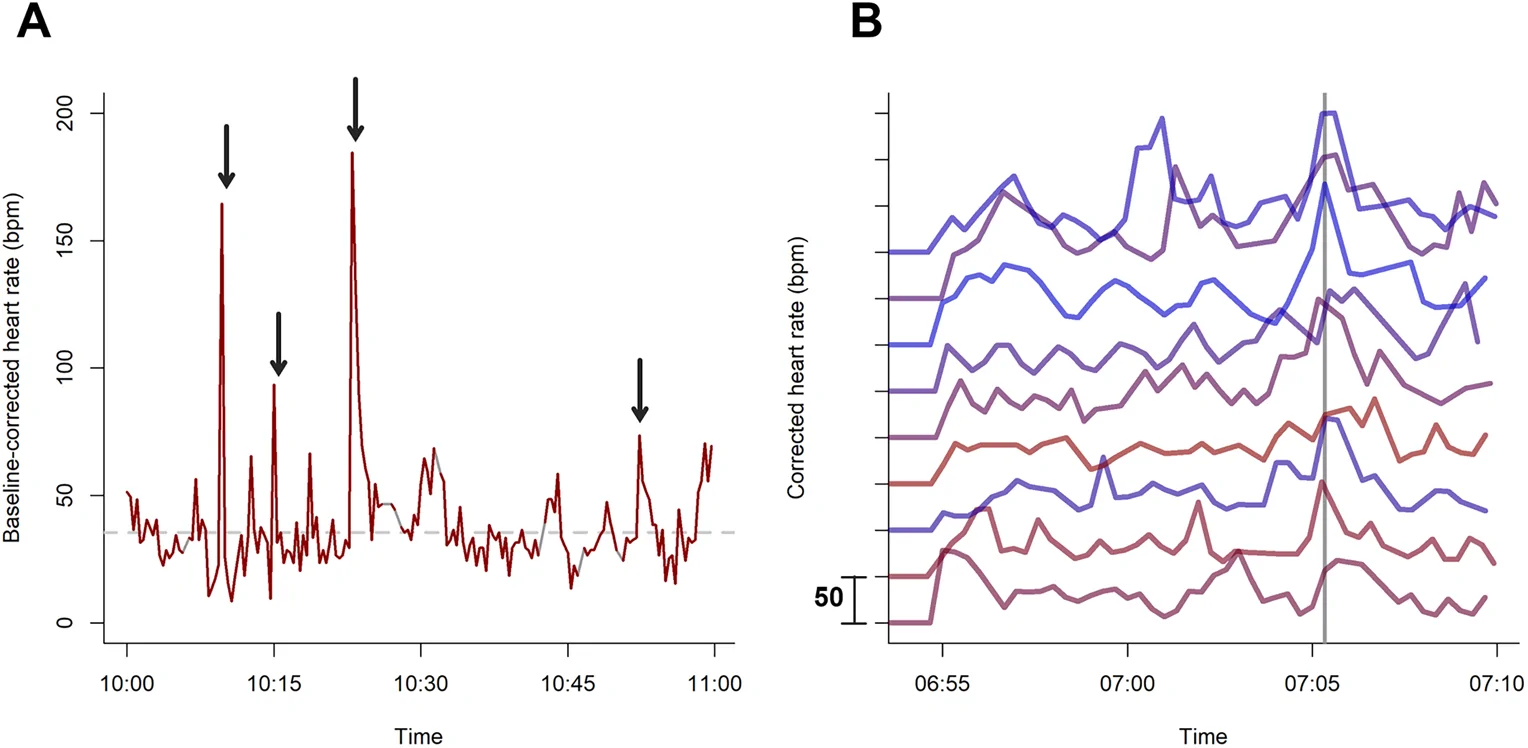
Detection of acute spikes in HR across a group of vulturine guineafowl. (A) Example of the data from an ECG logger implanted into one individual, showing daily median (dashed grey line) baseline-corrected HR with four detected spikes (arrows) in HR (red; gaps > 20 s in data, solid grey) over the course of 1 h. (B) Aligned data across nine individuals over 15 min, highlighting a collective response (vertical grey line) to a likely predation threat. Baseline-corrected HRs are shown starting from 0 (level line prior to 06:55), with *y*-axis ticks denoting a scale of 50 beats per minute (bpm).

To identify when multiple individuals were experiencing simultaneous spikes in HR, we first aligned all time windows into 20 s bins to account for misaligned bursts of data across individuals arising from ECG logger clock drift. This temporal misalignment of some data and cases where the raw data were filtered out as part of our data cleaning [22] means that the number of birds represented in each sampling window varied. We excluded all windows in which fewer than five individuals contributed data (whether spiking or not), as these were more likely to produce inaccurate estimates of the number of concurrent spikes in HR.

To distinguish between acute stressors arising from likely social versus likely predator sources, we used the number of individuals showing simultaneous HR spikes (figure 1B) in a given window. Windows with one individual spiking were classified as ‘*single*’. Windows with more than one, but fewer than half of the individuals showing HR spikes were classified as ‘*social*’ because social interactions in vulturine guineafowl—such as dominance interactions, displacements or aggressive chases—typically only involve a small subset of group members [17,31]. By contrast, ‘*predator*’ responses were defined as those where a notable majority (at least 50% of individuals and at least four individuals) had a HR spike in the same data window. We also labelled windows containing no HR spikes as ‘*normal*’, and labelled individuals that were not experiencing a HR spike in the same time window where at least one other individual was experiencing a spike as ‘*bystanders*’.

We interpret collective majority events as likely responses to acute predator-related threats. These threats likely included real predation attempts or alarm calls triggered by predator presence. There are justifications for this interpretation. (i) HR spikes are highly synchronous and transient (typically <20 s before HR returns to pre-spike levels), consistent with startle-type sympathetic activation characteristic of acute threat responses (i.e. immediate steep spike in HR caused by a sympathetic surge [10,32,33]) rather than sustained excitement or movement (i.e. lower-intensity or more-sustained increase in HR). (ii) Other potential causes of collective disturbance (e.g. vehicle noise, researcher presence and food competition) are rare at our remote field site and no food provisioning took place within the group's home range during this study. (iii) Coordinated group-level escape responses in vulturine guineafowl are almost exclusively observed in response to predator presence or alarm calls, and not during normal foraging, social activities or in response to human activities (personal observations). Thus, while some events may arise from other sources, the majority of collective spikes are likely to be predation-related.

### (d) Comparison to observed rates of agonistic interactions

To ground-truth our data, we compared rates of HR spikes attributed to social stressors to rates of aggressive social interactions taken from focal observations of two habituated guineafowl groups. These include the group tracked in this study (covering a period of 1 year prior to the start of tracking, as we did not collect observational data during ECG data collection) and a second group of comparable size (observations over 4 years, 2019–2023, with a mean group size of 29 individuals). We recorded agonistic interactions [17], using an all-occurrence sampling method [34], and calculated interaction rates (aggressive interactions per hour) on a *per-capita* basis. To account for the fact that HR spikes could only be collected from a subset of group members (and hence we would miss cases where one participant did not have an ECG logger), we multiplied the interaction rates derived from observational data by the proportion of group members present in the HR data.

### (e) Statistical analyses

We first confirmed that the collective spikes in HR we observed were unlikely to occur randomly owing to our large sample size. We addressed this by calculating the mean individual HR spike rate in our dataset (*r*, approx. 2.2% of time windows). We then estimated the binomial probability (prob*_s_*; equation (2.1)) of observing *s* simultaneous spikes (from 1 to *g*, where *g* is the number of individuals with ECG data in that window):

(2.1) $$prob_{s}=\left(\begin{array}{c} g\\ s \end{array}\right)r^{s}{\left(1-r\right)}^{g-s},$$

and multiplied the output probabilities by the number of time windows containing that many available individuals (*n*), giving us the expected number of observed instances (*λ*, where $\lambda \;=\;n\times prob_{s}$). Finally, we calculated the cumulative Poisson's probability (*P*) of observing *X* or more instances of *s* simultaneous spikes among *g* individuals equation (2.2):

(2.2) $$P=Pr(X^{\prime }\geq X\;|\lambda )=1-\overset{X-1}{\underset{k\;=\;0}{\sum }}\frac{\lambda^{k}e^{-\lambda }}{k!},$$

which we considered as non-random observations when *P* < 0.05.

To test for the effects of different types of spike events on individuals’ cardiac and behavioural responses, we constructed linear mixed-effects models. We fit two models, one testing whether individuals’ baseline-corrected HRs varied by event type, and a second model testing for movement responses to different event types—measured as acceleration or the change in mean speed (in m s^–1^ over each 20 s window) between two concurrent time windows (from the 1 Hz GPS data). In both models, event type was fit as a categorical predictor with ‘*normal*’ windows as the reference level and with individual identity as a random effect. We also conducted *post hoc* tests (comparing each event type to every other event type) based on pairwise contrasts of the estimated marginal means, derived using the R package ‘emmeans’ [35], for each model.

## 3. Results

Across 28 921 aligned data windows (179 460 individual measures of HR changes), representing 160.67 recording hours, we identified 4875 HR spikes (2.7% of all changes in HR). These included 22 collective responses to likely predation events (105 individual spikes, figure 2, which is significantly higher than expected by chance; supplementary material, table S1) and 549 likely social responses (1174 spikes comprising two to three individuals; figure 2). In cases where the group was spatially split (at least one dyad >135 m apart), all HR spikes (49 spikes across nine events) were expressed by individuals within the same subgroup, further confirming the collective nature of these events. On average, HR spikes corresponded to a 49.8 beats per minute (bpm.; s.d. = 28.2) increase, reaching an average baseline-corrected HR of 93.6 bpm (s.d. = 34.8; mean uncorrected HR = 237.4 bpm).

**Figure 2.**
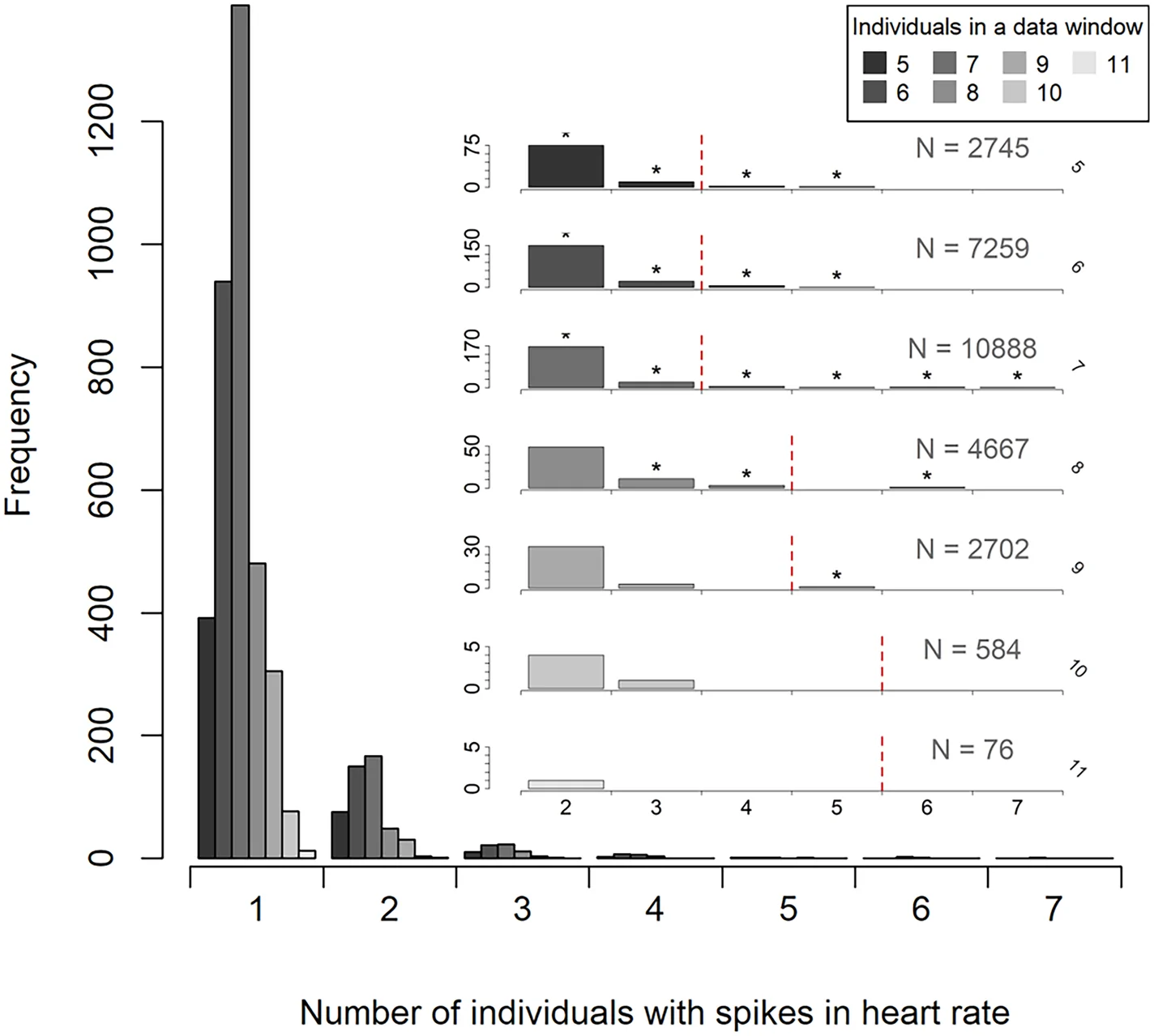
Distribution of group members with simultaneous spikes in HR per data window, across different numbers of individuals represented in the data. Subpanels show the tail of the distribution. Red dashed lines represent thresholds for the number of simultaneous spikes needed to define responses to likely predation events in data windows containing different numbers of birds (five to 11). Stars show where the number of simultaneous responses were recorded more frequently than expected by chance. Grey numbers represent sample sizes (total data windows where that number of birds have data).

From an individual's perspective, our results suggest an >11-fold higher rate of HR spikes in response to social stressors (median 0.72 per hour; supplementary material, table S2) than to predation threats (median 0.06 per hour). Our estimated rates of social interactions based on HR spikes were also identical to those derived from 5284 direct observations of aggressive interactions (median 0.72 per hour).

Despite being much rarer, we found that predation-related HR spikes elicit stronger behavioural and physiological responses. Birds accelerated twice as fast in response to likely predation events as to likely social events (figure 3A; supplementary material, table S3). Corrected HRs during responses to social stressors were, on average, 257% higher than during ‘normal’ non-spiking periods, but HRs from likely predation responses were a further 38% greater than social responses (nearly five times normal values, figure 3B; supplementary material, table S4). Across all contexts, increasing numbers of simultaneous spikes within the group led to increased corrected HR (approx. 18 bpm increase per additional spiking group member; supplementary material, table S5) and acceleration values (0.04 m s^–1^ per additional spike; supplementary material, table S5).

**Figure 3.**
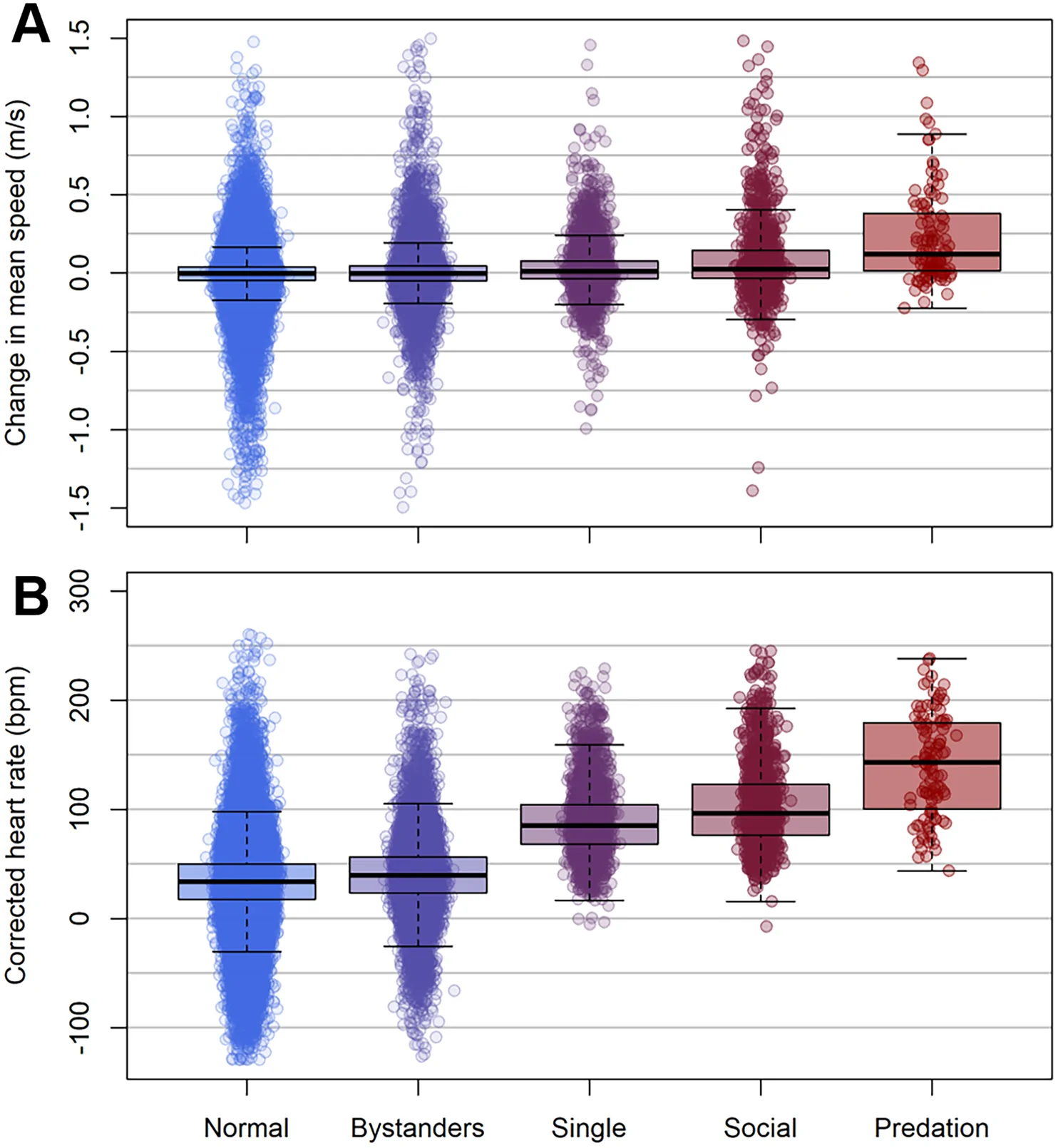
Behavioural and physiological responses following HR spikes are stronger for events involving more group members. (A) Changes in mean individual speed for individuals across different types of events. These include when no group member experienced a HR spike (normal), individuals not experiencing a spike in an event where at least one individual experienced a HR spike (bystanders), a sole individual experiencing a spike in a window (single), two to three individuals experiencing a spike in the same data window (likely social events) and collective spikes where at least 50% of individuals had a HR spike (likely predation events). (B) Peak in baseline-corrected HRs (bpm) for individuals in different types of events (per panel A). All pairwise contrasts in both panels are statistically significant at *p* < 0.05, in tables S3 and S4 in the supplementary material.

## 4. Discussion

Our results reveal that while reducing predation risk is widely considered a predominant driver of individual and group behaviour, these benefits are clearly traded off with increases in social sources of stress. Specifically, individual birds experienced >11 times as many HR spikes resulting from likely social events as they did from likely predator-related events (this ratio may be as high as 17 times within the range of reasonable thresholds for detecting spikes; supplementary material, figure S1). However, the intensity of responses to likely social events was lower than to likely predation-related events. While we found that birds accelerate faster in response to likely predator events, the threat of predation itself may induce additional physiological stress above and beyond the costs of movement. Guineafowl typically run (often flapping) towards the nearest vegetation at the sound of an aerial alarm call [23], whereas few agonistic interactions result in extended chases [17]. Thus, we find support for the predictions that predator-related events elicit stronger responses, while socially driven events are much more frequent.

While our dataset represented a subset of group members and we cannot determine the contexts of events with certainty, our estimation of rates of social stressors versus predation threats is unlikely to be biased towards the social data. This is because we are more likely to detect likely predation events (as they affect most group members) than we are to detect social events (because they might affect individuals that do not have a logger). Further, our inferred rate of social events from the HR data (0.72 events per individual per hour) was identical to the rate calculated from observational data, suggesting our thresholds for assigning spikes were reasonable (see supplementary material, figure S1 for alternative thresholds). These rates are also consistent with those found for individuals in other group-living species (e.g. mean of 0.61 per female among non-human primates [36], 0.76–1.46 per hour in male Przewalski horses *Equus przewalskii* [37,38]). Together, these different lines of evidence suggest that our findings are robust.

One key question is whether our relative rates of social versus predator-related events (i.e. the 11-fold difference) are general across species and contexts. Our study was started at the onset of one of the worst recorded droughts in East Africa. In fact, we did not observe any reproduction in the population (including our focal group) between 2020 and 2023. Previous work in this population [17] found that rates of agonistic interaction increase substantially during wetter conditions (when birds have a chance to breed). By contrast, predation rates are much higher during dry periods (DR Farine 2016–2026, unpublished data), likely because birds spend substantially more time foraging, including during riskier hours of the day [27]. These patterns are consistent with many other species, for example, juvenile ungulates have higher survival under higher rainfall [39]. Thus, it is likely that our data reflect the lower end of this difference, and that the relative rate of social versus predation events should be much higher when conditions are better, and especially when breeding is likely to take place.

Forming groups introduces novel trade-offs for animals. While group living provides safety from predators [40], it requires animals to overcome many new challenges. These include resolving differences in preferences about where to go [41,42] and navigating complex dominance hierarchies [43]. More recently, there has been greater acknowledgement of some of the physiological costs associated with group living [44,45], such as reduced movement efficiency [46], resolving conflicting preferences [22] and the costs associated with agonistic social interactions [18,19]. Our findings suggest that the latter are likely to be far more common than classical ecological costs, like predation. This highlights an understudied evolutionary trade-off in social animals, where reducing predation risks comes at the cost of frequent lower-intensity social stressors, introducing a new lens through which we may be able to understand constraints on group sizes.

## Supplementary Material



## Data Availability

Data and code used in this study are available on the Figshare repository [47]. Supplementary material is available online [48].

## References

[1] Rubenstein DI . 1978 On predation, competition, and the advantages of group living. Perspect. Ethol. 3, 205–231. (doi:10.1007/978-1-4684-2901-5_9)

[2] Hamilton WD . 1971 Geometry for the selfish herd. J. Theor. Biol. 31, 295–311. (doi:10.1016/0022-5193(71)90189-5) 5104951

[3] Foster WA , Treherne JE. 1981 Evidence for the dilution effect in the selfish herd from fish predation on a marine insect. Nature 293, 466–467. (doi:10.1038/293466a0)

[4] Lima SL . 1995 Back to the basics of anti-predatory vigilance: the group-size effect. Anim. Behav. 49, 11–20. (doi:10.1016/0003-3472(95)80149-9)

[5] Roberts G . 1996 Why individual vigilance declines as group size increases. Anim. Behav. 51, 1077–1086. (doi:10.1006/anbe.1996.0109)

[6] Landeau L , Terborgh J. 1986 Oddity and the ‘confusion effect’ in predation. Anim. Behav. 34, 1372–1380. (doi:10.1016/S0003-3472(86)80208-1)

[7] Palmer MS , Gaynor KM, Becker JA, Abraham JO, Mumma MA, Pringle RM. 2022 Dynamic landscapes of fear: understanding spatiotemporal risk. Trends Ecol. Evol. 37, 911–925. (doi:10.1016/j.tree.2022.06.007) 35817684

[8] Hawlena D , Schmitz OJ. 2010 Physiological stress as a fundamental mechanism linking predation to ecosystem functioning. Am. Nat. 176, 537–556. (doi:10.1086/656495) 20846014

[9] Cyr NE , Dickens MJ, Romero LM. 2009 Heart rate and heart-rate variability responses to acute and chronic stress in a wild-caught passerine bird. Physiol. Biochem. Zool. 82, 332–344. (doi:10.1086/589839) 19115847

[10] Carravieri A , Müller MS, Yoda K, Hayama S, Yamamoto M. 2016 Dominant parasympathetic modulation of heart rate and heart rate variability in a wild-caught seabird. Physiol. Biochem. Zool. 89, 263–276. (doi:10.1086/686894) 27327178

[11] Chabot D , Gagnon P, Dixon EA. 1996 Effect of predator odors on heart rate and metabolic rate of wapiti (*Cervus elaphus canadensis*). J. Chem. Ecol. 22, 839–868. (doi:10.1007/BF02033590) 24227589

[12] Janczarek I , Stachurska A, Kędzierski W, Wiśniewska A, Ryżak M, Kozioł A. 2020 The intensity of physiological and behavioral responses of horses to predator vocalizations. BMC Vet. Res. 16, 431. (doi:10.1186/s12917-020-02643-6) PMC7653799 33167961

[13] Lima SL , Dill LM. 1990 Behavioral decisions made under the risk of predation: a review and prospectus. Can. J. Zool. 68, 619–640. (doi:10.1139/z90-092)

[14] Culik B , Adelung D, Woakes AJ. 1990The effect of disturbance on the heart rate and behaviour of Adélie penguins (*Pygoscelis adeliae*) during the breeding season. In Antarctic ecosystems (eds KR Kerry, G Hempel), pp. 177–182. Berlin, Germany: Springer.

[15] Ellenberg U , Mattern T, Houston DM, Davis LS, Seddon PJ. 2012 Previous experiences with humans affect responses of Snares penguins to experimental disturbance. J. Ornithol. 153, 621–631. (doi:10.1007/s10336-011-0780-4)

[16] Nephew BC , Romero LM. 2003 Behavioral, physiological, and endocrine responses of starlings to acute increases in density. Horm. Behav. 44, 222–232. (doi:10.1016/j.yhbeh.2003.06.002) 14609544

[17] Dehnen T , Papageorgiou D, Nyaguthii B, Cherono W, Penndorf J, Boogert NJ, Farine DR. 2022 Costs dictate strategic investment in dominance interactions. Phil. Trans. R. Soc. B 377, 20200447. (doi:10.1098/rstb.2020.0447) PMC8743880 35000443

[18] Wascher CAF , Scheiber IBR, Weiß BM, Kotrschal K. 2009 Heart rate responses to agonistic encounters in greylag geese, *Anser anser*. Anim. Behav. 77, 955–961. (doi:10.1016/j.anbehav.2009.01.013)

[19] Wascher CAF , Scheiber IBR, Kotrschal K. 2008 Heart rate modulation in bystanding geese watching social and non-social events. Proc. R. Soc. B 275, 1653–1659. (doi:10.1098/rspb.2008.0146) PMC2602813 18430645

[20] Brandl HB , Pruessner JC, Farine DR. 2022 The social transmission of stress in animal collectives. Proc. R. Soc. B 289, 20212158. (doi:10.1098/rspb.2021.2158) PMC9091854 35538776

[21] Marchant JN , Mendl MT, Rudd AR, Broom DM. 1995 The effect of agonistic interactions on the heart rate of group-housed sows. Appl. Anim. Behav. Sci. 46, 49–56. (doi:10.1016/0168-1591(95)00636-2)

[22] Brandl HB *et al.* 2025 The physiological cost of leadership in collective movements. Curr. Biol. 35, 4003–4010. (doi:10.1016/j.cub.2025.06.065) 40701152

[23] van Niekerk JH , Forcina G, Megía-Palma R. 2024 Grouping behaviour and anti-predator responses in the helmeted guineafowl *Numida meleagris*. Birds 5, 685–702. (doi:10.3390/birds5040047)

[24] Papageorgiou D , Christensen C, Gall GEC, Klarevas-Irby JA, Nyaguthii B, Couzin ID, Farine DR. 2019 The multilevel society of a small-brained bird. Curr. Biol. 29, R1120–R1121. (doi:10.1016/j.cub.2019.09.072) 31689393

[25] Nyaguthii B , Dehnen T, Klarevas-Irby JA, Papageorgiou D, Kosgey J, Farine DR. 2025 Cooperative and plural breeding by the precocial vulturine guineafowl. Ibis 165, 695–710. (doi:10.1111/ibi.13393)

[26] de Boer T , Sikenykeny K, Nyaguthii B, Farine DR, Klarevas-Irby JA. 2025 Habitat selection during dispersal reduces the energetic cost of transport when making large displacements. Proc. R. Soc. B 292, 20251442. (doi:10.1098/rspb.2025.1442) PMC12646769 41290165

[27] Christensen C , Sikenykeny K, Farine DR. 2025 Resource scarcity increases foraging activity despite thermal risk in an arid-adapted bird. bioRxiv. (doi:10.1101/2025.11.13.688088)

[28] Klarevas‐Irby JA , Wikelski M, Farine DR. 2021 Efficient movement strategies mitigate the energetic cost of dispersal. Ecol. Lett. 24, 1432–1442. (doi:10.1111/ele.13763) 33977638

[29] Papageorgiou D , Nyaguthii B, Farine DR. 2024 Compromise or choose: shared movement decisions in wild vulturine guineafowl. Commun. Biol. 7, 95. (doi:10.1038/s42003-024-05782-w) PMC10787764 38218910

[30] He P , Klarevas‐Irby JA, Papageorgiou D, Christensen C, Strauss ED, Farine DR. 2023 A guide to sampling design for GPS-based studies of animal societies. Methods Ecol. Evol. 14, 1887–1905. (doi:10.1111/2041-210X.13999)

[31] Dehnen T , Nyaguthii B, Cherono W, Boogert NJ, Farine DR. 2025 Breeding-related changes in social interactions among female vulturine guineafowl. Ecol. Evol. 15, e70943. (doi:10.1002/ece3.70943) PMC11783233 39896772

[32] Weimerskirch H , Shaffer SA, Mabille G, Martin J, Boutard O, Rouanet JL. 2002 Heart rate and energy expenditure of incubating wandering albatrosses: basal levels, natural variation, and the effects of human disturbance. J. Exp. Biol. 205, 475–483. (doi:10.1242/jeb.205.4.475) 11893761

[33] Kanwisher JW , Williams TC, Teal JM, Lawson KO. 1978 Radiotelemetry of heart rates from free-ranging gulls. Auk 95, 288–293. https://digitalcommons.usf.edu/auk/vol95/iss2/7?utm_source=digitalcommons.usf.edu%2Fauk%2Fvol95%2Fiss2%2F7&utm_medium=PDF&utm_campaign=PDFCoverPages.

[34] Altmann J . 1974 Observational study of behavior: sampling methods. Behaviour 49, 227–266. (doi:10.1163/156853974X00534) 4597405

[35] Lenth R , Piaskowski J. 2025 Emmeans: estimated marginal means, aka least-squares means. R package version 2.0.0. (doi:10.32614/CRAN.package.emmeans)

[36] Wheeler BC , Scarry CJ, Koenig A. 2013 Rates of agonism among female primates: a cross-taxon perspective. Behav. Ecol. 24, 1369–1380. (doi:10.1093/beheco/art076) PMC3796709 24137045

[37] Feh C . 1988 Social behaviour and relationships of Przewalski horses in Dutch semi-reserves. Appl. Anim. Behav. Sci. 21, 71–87. (doi:10.1016/0168-1591(88)90101-3)

[38] Christensen JW , Zharkikh T, Ladewig J, Yasinetskaya N. 2002 Social behaviour in stallion groups (*Equus przewalskii* and *Equus caballus*) kept under natural and domestic conditions. Appl. Anim. Behav. Sci. 76, 11–20. (doi:10.1016/S0168-1591(01)00208-8)

[39] Owen‐smith N , Mason DR, Ogutu JO. 2005 Correlates of survival rates for 10 African ungulate populations: density, rainfall and predation. J. Anim. Ecol. 74, 774–788. (doi:10.1111/j.1365-2656.2005.00974.x)

[40] Krause J , Ruxton G. 2002The benefits of group formation. In Living in groups. pp. 6–40. Oxford, UK: Oxford University Press. (doi:10.1093/oso/9780198508175.003.0002)

[41] Conradt L , Roper TJ. 2005 Consensus decision making in animals. Trends Ecol. Evol. 20, 449–456. (doi:10.1016/j.tree.2005.05.008) 16701416

[42] Strandburg-Peshkin A , Farine DR, Couzin ID, Crofoot MC. 2015 Shared decision-making drives collective movement in wild baboons. Science 348, 1358–1361. (doi:10.1126/science.aaa5099) PMC4801504 26089514

[43] Strauss ED , Curley JP, Shizuka D, Hobson EA. 2022 The centennial of the pecking order: current state and future prospects for the study of dominance hierarchies. Phil. Trans. R. Soc. B 377, 20200432. (doi:10.1098/rstb.2020.0432) PMC8743894 35000437

[44] Seebacher F , Krause J. 2017 Physiological mechanisms underlying animal social behaviour. Phil. Trans. R. Soc. B 372, 20160231. (doi:10.1098/rstb.2016.0231) PMC5498293 28673909

[45] Wascher CAF . 2021 Heart rate as a measure of emotional arousal in evolutionary biology. Phil. Trans. R. Soc. B 376, 20200479. (doi:10.1098/rstb.2020.0479) PMC8237168 34176323

[46] Klarevas-Irby JA , Nyaguthii B, Farine DR. 2025 Moving as a group imposes constraints on the energetic efficiency of movement. Proc. R. Soc. B 292, 20242760. (doi:10.1098/rspb.2024.2760) PMC11836700 39968615

[47] Klarevas-Irby J , Brandl HB, Farine DR. 2026 Data and code for ‘Collective heart rate telemetry reveals frequent cardiac responses to social interactions but stronger responses to predation. Figshare. (doi:10.6084/m9.figshare.33994654) PMC13752844 43012321

[48] Klarevas-Irby JA , Brandl HB, Farine DR. 2026 Supplementary material from: Collective heart rate telemetry reveals frequent cardiac responses to social interactions but stronger responses to predation. Figshare. (doi:10.6084/m9.figshare.c.8737324) PMC13752844 43012321

