# Supplementary material for: Collective heart rate telemetry reveals frequent cardiac responses to social interactions but stronger responses to predation
Source: Biol Lett. 2026 Nov 11;22(11):20260478. doi: 10.1098/rsbl.2026.0478 (PMC13752844; doi:10.1098/rsbl.2026.0478)
Supplement: Supplementary file 1 [file rsbl.2026.0478.zip › rsbl.2026.0478/rsbl20260478_si_001.docx]

**Supplementary materials for: Collective heart rate telemetry reveals frequent cardiac responses to social interactions but stronger responses to predation**

James A. Klarevas-Irby^1,3,4,7^ & Hanja B. Brandl^2,5,7^, Damien R. Farine^2,3,6,8,^*

^1^Department of Migration, Max Planck Institute of Animal Behavior, 78315 Radolfzell, Germany.

^2^Department of Collective Behavior, Max Planck Institute of Animal Behavior, 78646 Konstanz, Germany.

^3^Division of Ecology and Evolution, Research School of Biology, Australian National University, Canberra 2601, Australia.

^4^Mpala Research Centre, Nanyuki 10400, Kenya.

^5^Centre for the Advanced Study of Collective Behaviour, University of Konstanz, 78464 Konstanz, Germany.

^6^Department of Ornithology, National Museums of Kenya, 00100 Nairobi, Kenya.

^7^These authors contributed equally to this work and are joint first author.

^8^Lead contact.

*Correspondence: [Damien.Farine@anu.edu.au](mailto:Damien.Farine@anu.edu.au)

**Supplementary Figure S1. Impact of alternative thresholding of changes in HR as used to assign HR spikes.** (A) Variation in the detected frequency of assigned social (blue) and predation (red) responses, as measured by the mean hourly rate at which any given individual experienced a spike due to either stressor. Dashed blue line shows the rate of social interactions as derived from direct observations of two comparably sized guineafowl groups. Values above points show the ratio of assigned social:predation response rates. Arrow indicates the quantile used for all analyses (95^th^ quantile). (B) Magnitude of HR responses to likely social (blue) and predation (red) events, as measured by the baseline-corrected HR following an assigned HR spike, across different threshold quantiles. Values above pairs of boxes show the difference in median HR values (predation-social) at each threshold, with larger values indicating stronger relative responses to likely predation events.

Supplementary Tables

**Supplementary Table S1.** Numbers of events with a given number of simultaneous HR spikes from a given number of available individuals. Values shown only represent cases which were observed in our dataset, and not all possible cases which could have been observed (e.g. no cases of 5 simultaneous spikes out of 8 available individuals were recorded). P values represent the cumulative Poisson probability of observing at least as many events as present in our data for each given sample size and number of simultaneous HR spikes. These data correspond to Figure 2 in the main text.

| **Probability that observed simultaneous HR spikes were non-random** | | | | |
| --- | --- | --- | --- | --- |
| Number of individuals present in data (N) | Data windows with N individuals present | Individuals with simultaneous HR spikes (*n*) | Observed events with *n* simultaneous HR spikes | P |
| **5** | 2,745 | 1 | 392 | **<0.001** |
|  |  | 2 | 75 | **<0.001** |
|  |  | 3 | 10 | **<0.001** |
|  |  | 4 | 2 | **<0.001** |
|  |  | 5 | 1 | **<0.001** |
| **6** | 7,259 | 1 | 939 | **0.010** |
|  |  | 2 | 150 | **<0.001** |
|  |  | 3 | 22 | **<0.001** |
|  |  | 4 | 7 | **<0.001** |
|  |  | 5 | 1 | **<0.001** |
| **7** | 10,888 | 1 | 1,390 | 0.995 |
|  |  | 2 | 167 | **<0.001** |
|  |  | 3 | 23 | **<0.001** |
|  |  | 4 | 6 | **<0.001** |
|  |  | 5 | 1 | **0.001** |
|  |  | 6 | 2 | **<0.001** |
|  |  | 7 | 1 | **<0.001** |
| **8** | 4,667 | 1 | 481 | 1.000 |
|  |  | 2 | 49 | 0.872 |
|  |  | 3 | 11 | **<0.001** |
|  |  | 4 | 3 | **<0.001** |
|  |  | 6 | 1 | **<0.001** |
| **9** | 2,702 | 1 | 305 | 1.000 |
|  |  | 2 | 30 | 0.974 |
|  |  | 3 | 3 | 0.381 |
|  |  | 5 | 1 | **0.002** |
| **10** | 584 | 1 | 77 | 0.999 |
|  |  | 2 | 4 | 0.995 |
|  |  | 3 | 1 | 0.487 |
| **11** | 76 | 1 | 12 | 0.809 |
|  |  | 2 | 1 | 0.818 |

**Supplementary Table S2.** Summary of per-individual occurrences of HR spike events of each category, total number of 20-second data windows, and corresponding total hours of data available per individual used to calculate the rates at which individuals experience different potential stressors.

|  | **Event type** | | | | |  |  |
| --- | --- | --- | --- | --- | --- | --- | --- |
| **Individual** | **Normal** | **Bystander** | **Single** | **Social** | **Environmental** | **Total windows** | **Total hours tracked** |
| **W1298** | 762 | 91 | 9 | 5 | 2 | 893 | 4.96 |
| **W1307** | 19641 | 2860 | 356 | 118 | 11 | 23440 | 130.22 |
| **W1312** | 21938 | 2869 | 515 | 192 | 13 | 26071 | 144.84 |
| **W1394** | 22526 | 3063 | 456 | 177 | 17 | 26624 | 147.91 |
| **W1543** | 22466 | 3005 | 519 | 162 | 20 | 26656 | 148.09 |
| **W1544** | 21463 | 2995 | 350 | 129 | 10 | 25705 | 142.81 |
| **W1546** | 2654 | 392 | 24 | 2 | 0 | 3101 | 17.23 |
| **WT00162** | 5917 | 786 | 112 | 11 | 0 | 6914 | 38.41 |
| **WT00493** | 22408 | 2964 | 507 | 168 | 16 | 26473 | 147.07 |
| **WT00584** | 21339 | 2782 | 529 | 169 | 13 | 25344 | 140.80 |
| **WT00599** | 9094 | 1007 | 219 | 41 | 3 | 10604 | 58.91 |

**Supplementary Table S3.** Results of the LMM for acceleration (change in mean speed over 20s between consecutive time windows) and its response to categorical context (i.e. normal, bystander to a spike event, single individual immediately following an HR spike, social spike event, or environmental spike event), with individual identity fit as a random effect. Reference level of contexts is normal (no group member exhibiting a spike). All pairwise posthoc tests were significant at P<0.001 (results not shown). The data for these model results are shown in Figure 3A.

| **Acceleration following a HR spike event** | | | | | | | | | | |
| --- | --- | --- | --- | --- | --- | --- | --- | --- | --- | --- |
| **Equation:** Acceleration *~ Context + (1\|Individual ID)* | | | | | | | | | | |
|  | | **Estimate** | | **Std. Error** | | | **Df** | **t value** | | **Pr(>\|t\|)** |
| **Intercept (Normal)** | | -4.173 e-03 | | 5.170 e-04 | | | 6.850 | -8.071 | | **<0.001** |
| **Bystander** | | 6.235 e-03 | | 9.834 e-04 | | | 1.792 e+05 | 6.341 | | **<0.001** |
| **Single** | | 3.591 e-02 | | 2.128 e-03 | | | 1.794 e+05 | 16.876 | | **<0.001** |
| **Social** | | 1.002 e-01 | | 3.695 e-03 | | | 1.794 e+05 | 27.108 | | **<0.001** |
| **Environmental** | | 2.352 e-1 | | 1.231 e-02 | | | 1.794 e+05 | 19.105 | | **<0.001** |
| ***Random effects*** | | | | | | | | | | |
| **Groups** | **Name** | | **Mean Variance** | | | **Std.dev** | |  |  |  |
| **Individual ID** | **Intercept** | | 1.496 e-06 | | | 0.001 | |  |  |  |
| **Residual** |  | | 1.591 e-02 | | | 0.126 | |  |  |  |
| **Marginal r^2^** | | | | | **Conditional r^2^** | | | | | |
| 0.008 | | | | | 0.008 | | | | | |

**Supplementary Table S4.** Results of the LMM for baseline-corrected heart rate (bpm) and its response to categorical context (i.e. normal, bystander to a spike event, single individual immediately following an HR spike, social spike event, or environmental spike event), with individual identity fit as a random effect. Reference level of contexts is ‘normal’ (no group member exhibiting a spike). All pairwise posthoc tests were significant at P<0.001 (results not shown). The data for these model results are shown in Figure 3B.

| **Heart Rate following a HR spike event** | | | | | | | | | | | |
| --- | --- | --- | --- | --- | --- | --- | --- | --- | --- | --- | --- |
| **Equation:** HR_corrected_ *~ Context + (1\|Individual ID)* | | | | | | | | | | | |
|  | | **Estimate** | | **Std. Error** | | | **Df** | | **t value** | | **Pr(>\|t\|)** |
| **Intercept (Normal)** | | 26.55 | | 5.272 | | | 9.998 e+00 | | 5.036 | | **<0.001** |
| **Bystander** | | 6.67 | | 1.954 e-01 | | | 1.979 e+05 | | 34.159 | | **<0.001** |
| **Single** | | 54.48 | | 4.457 e-01 | | | 1.979 e+05 | | 122.239 | | **<0.001** |
| **Social** | | 68.35 | | 7.746 e-01 | | | 1.979 e+05 | | 88.235 | | **<0.001** |
| **Environmental** | | 104.72 | | 2.582 | | | 1.979 e+05 | | 40.567 | | **<0.001** |
| ***Random effects*** | | | | | | | | | | | |
| **Groups** | **Name** | | **Mean Variance** | | | **Std.dev** | |  | |  |  |
| **Individual ID** | **Intercept** | | 305.6 | | | 17.48 | |  | |  |  |
| **Residual** |  | | 699.4 | | | 26.45 | |  | |  |  |
| **Marginal r^2^** | | | | | **Conditional r^2^** | | | | | | |
| 0.08 | | | | | 0.36 | | | | | | |

**Supplementary Table S5.** Results of two LMMs for baseline-corrected heart rate (bpm) and acceleration as they each respond to the number of group members exhibit (i.e. irrespective of spike context), with individual identity fit as a random effect.

| **Heart Rate response to the number of concurrent HR spikes within the group** | | | | | | | | | | | |
| --- | --- | --- | --- | --- | --- | --- | --- | --- | --- | --- | --- |
| **Equation:** HR_corrected_ *~* N_spiking_ *+ (1\|Individual ID)* | | | | | | | | | | | |
|  | | **Estimate** | | **Std. Error** | | | **Df** | | **t value** | | **Pr(>\|t\|)** |
| **Intercept** | | 22.30 | | 6.236 | | | 1.012 e+01 | | 3.576 | | **0.005** |
| **N_spiking_** | | 17.98 | | 2.582 | | | 1.979 e+05 | | 40.567 | | **<0.001** |
| ***Random effects*** | | | | | | | | | | | |
| **Groups** | **Name** | | **Mean Variance** | | | **Std.dev** | |  | |  |  |
| **Individual ID** | **Intercept** | | 423.2 | | | 20.57 | |  | |  |  |
| **Residual** |  | | 1281.6 | | | 35.80 | |  | |  |  |
| **Marginal r^2^** | | | | | **Conditional r^2^** | | | | | | |
| 0.04 | | | | | 0.28 | | | | | | |
|  | | | | |  | | | | | | |
| **Acceleration in response to the number of concurrent HR spikes within the group** | | | | | | | | | | | |
| **Equation:** Acceleration *~* N_spiking_ *+ (1\|Individual ID)* | | | | | | | | | | | |
|  | | **Estimate** | | **Std. Error** | | | **Df** | | **t value** | | **Pr(>\|t\|)** |
| **Intercept** | | -3.614 e-02 | | 2.883 e-03 | | | 1.690 e+02 | | -12.53 | | **<0.001** |
| **N_spiking_** | | 4.070 e-02 | | 2.225 e-03 | | | 2.531 e+04 | | 18.30 | | **<0.001** |
| ***Random effects*** | | | | | | | | | | | |
| **Groups** | **Name** | | **Mean Variance** | | | **Std.dev** | |  | |  |  |
| **Individual ID** | **Intercept** | | 4.209 e-06 | | | 0.002 | |  | |  |  |
| **Residual** |  | | 2.862 e-02 | | | 0.169 | |  | |  |  |
| **Marginal r^2^** | | | | | **Conditional r^2^** | | | | | | |
| 0.013 | | | | | 0.013 | | | | | | |
